# Mean Platelet Volume as a Marker of Vasculogenic Erectile Dysfunction and Future Cardiovascular Risk

**DOI:** 10.3390/jcm9082513

**Published:** 2020-08-04

**Authors:** Andrea Crafa, Rosita A. Condorelli, Laura M. Mongioì, Rossella Cannarella, Federica Barbagallo, Antonio Aversa, Giulia Izzo, Anna Perri, Aldo E. Calogero, Sandro La Vignera

**Affiliations:** 1 Department of Clinical and Experimental Medicine, University of Catania, via S. Sofia 78, 95123 Catania, Italy; crafa.andrea@outlook.it (A.C.); rosita.condorelli@unict.it (R.A.C.); lauramongioi@hotmail.it (L.M.M.); rossella.cannarella@phd.unict.it (R.C.); federica.barbagallo11@gmail.com (F.B.); acaloger@unict.it (A.E.C.); 2 Department of Experimental and Clinical Medicine, Magna Græcia University, 88100 Catanzaro, Italy; aversa@unicz.it (A.A.); giulia_izzo@unicz.it (G.I.); 3 Kidney and Transplantation Research Center, Department of Nephrology, Dialysis and Transplantation, Annunziata Hospital, 87100 Cosenza, Italy; perria71@gmail.com

**Keywords:** mean platelet volume, arteriogenic erectile dysfunction, coronary artery disease, vascular diseases, atherosclerosis

## Abstract

Cardiovascular diseases are the main cause of mortality in the Western population, so the attempt to find a marker capable of predicting their early onset is not surprising. It is known that arteriogenic erectile dysfunction (ED) precedes the onset of a major coronary event by several years. However, a marker that is able to early identify those patients who should undergo further diagnostic investigations is, to date, missing. Recent research on this topic has focused on the role of the mean platelet volume (MPV), a marker of platelet activity that is high in most vascular diseases, such as coronary artery disease (CAD), stroke, peripheral artery disease (PAD), and ED. The basic pathophysiological mechanism of all these clinical conditions is atherosclerosis. Platelets play a central role in amplifying this process both indirectly by stimulating endothelial cells to produce inflammatory cytokines and chemokines, and directly through the expression of membrane receptors and the release of molecules that contribute to the formation of atherosclerotic plaque. The objective of this review is to critically analyze the evidence on the role of MPV in predicting the diagnosis and severity of vasculogenic ED and the possibility of using this simple marker as a first step to start a diagnostic process aimed at assessing the cardiovascular risk in these patients.

## 1. Introduction

Erectile dysfunction (ED) is a very frequent symptom afflicting the male population. The average prevalence of the disorder is around 30% and grows with increasing age. In 80% of cases, it is associated with endocrine and non-endocrine organic causes. Among the latter, the vascular form is the one with the highest prevalence [1].

As is well known, arteriogenic ED is closely associated with cardiovascular risk. Indeed, 42–57% of patients with coronary artery disease (CAD) experience ED [2]. ED precedes the onset of a coronary event by at least three years, thus it is an early marker of cardiovascular risk [3]. This is probably due to the smaller diameter of the cavernous arteries compared to the coronary arteries (1–2 vs. 3–4 mm). Being the two conditions expression of the same atherosclerotic disease, the penile vascular bed is blocked before the coronary one [3,4]. Accordingly, it is not a coincidence that ED and CAD share the same risk factors, such as hypertension, smoking, dyslipidemia, obesity, diabetes, and thyroid dysfunction [5].

On this basis, the identification of markers of arteriogenic ED and, hence, of cardiovascular diseases (CVD) is useful. Several studies with this aim have focused on the role of the mean platelet volume (MPV) [6]. Therefore, this review aims to critically evaluate the evidence on the role of MPV in patients with ED and therefore the possible role of this marker in the stratification of the cardiovascular risk of these patients.

## 2. Role of the Platelets in the Pathogenesis of Atherosclerosis

Platelets are anucleated disc-shaped fragments derived from megakaryocytes, playing a fundamental role in the hemostatic process. However, the same mechanisms underlying their actions have been called into account as possible harmful factors in vascular diseases, including ED and CVD [7]. Indeed, platelets play a central role in the atherothrombotic process being able, once activated, to promote the development of chronic atherosclerotic lesions [8]. The first step for platelet activation is the reduced production by the dysfunctional endothelium of nitric oxide and prostacyclin, two molecules that normally contribute to platelet inhibition [9]. Activated platelets produce a series of inflammatory and mitogen mediators that further impair the endothelial function. Among these, a pivotal role is played by the CD40 ligand which in turn induces endothelial cells to produce reactive oxygen species (ROS), adhesion molecules, chemokines, and tissue factors with a consequent inflammatory response. The role of the CD40 ligand also appears to be confirmed by its increase in conditions typically associated with cardiovascular risk and atherosclerosis, such as diabetes mellitus and cigarette smoke [8,10]. Activated platelets also seem to produce a greater quantity of interleukin 1β, which stimulates endothelial cells to produce chemokines that facilitate the adhesion of neutrophils and monocytes at the endothelial level, thus promoting inflammation [8]. The recruitment of leukocytes in the vessel is also stimulated by mediators directly produced by platelets. Among these, the regulated upon activation, normal T cell expressed and secreted (RANTES) seems to be involved in the arrest of monocytes at the level of the inflamed and atherosclerotic endothelium [10,11]. Another important mediator is the platelet factor 4 (PF4), which favors the transformation of monocytes into macrophages and the deposition of low-density lipoproteins in the vessel. These events lead to the formation of foamy cells, which play a central role in the formation of the atherosclerotic lesion [11].

Activated platelets also express large quantities of P-selectin that binds neutrophils and monocytes, inducing their activation and promoting their transmigration across the endothelium [10,12]. Finally, a role also seems to be played by the vitronectin receptor (αVβ3) expressed on the platelet membrane. This acts by promoting the adhesion of platelets to the damaged vascular endothelium by binding with osteopontin, present in the atherosclerotic plaques, with consequent platelets activation [13]. Accordingly, in a previous study, we found that patients with arteriogenic ED express higher levels of αVβ3, suggesting a role for platelet activation in the etiopathogenesis of ED [14]. As further evidence of the relevance of the overexpression of this receptor in patients with ED, we found that the daily administration of tadalafil decreases the expression of αVβ3 and endothelial apoptotic microparticles (another important marker of endothelial damage), resulting in a decrease in platelet hyperactivity [15]. Hence, platelets represent the link between inflammation and thrombosis, the two fundamental processes underlying atherogenesis [11].

On this basis, the measurement of MPV is useful because platelet size predicts their activity. Indeed, several studies have shown that larger platelets synthesize more thromboxane A2 and B2 (the most potent vasoconstrictor agents) and express greater quantities of von Willebrand factor (VWF), fibrinogen, and P-selectin as well as a greater quantity of dense granules than smaller ones. They also produce a greater quantity of platelet-derived growth factors, which contribute to vascular neointimal proliferation. Finally, they are much more sensitive to ADP-induced aggregation and less sensitive to the antiplatelet action of prostacyclin. Consequently, larger platelets are more active and therefore more thrombogenic [16].

## 3. Mean Platelet Volume and Vascular Diseases

### 3.1. Arterial Diseases

Several studies have evaluated the role of MPV in CAD. A meta-analysis of 40 observational studies showed that patients with CAD on average have platelets 0.7 fl bigger than healthy subjects and an odds ratio of approximately 2.28 times of developing CAD in patients with high MPV compared to those with low MPV. The study also showed that the average increase in platelet size is greater in acute CAD (e.g., myocardial infarction) than in stable CAD (e.g., chronic stable angina). Finally, patients with low coronary blood flow have a higher MPV than those with a normal one [17]. Another study showed a reduced flow in the infarcted artery, before angioplasty, in patients with higher MPV values than those who had lower values. In this same study, infarcted patients with higher MPV had a greater risk of 30-day mortality than their counterparts with normal MPV [18]. Similarly, a meta-analysis of 16 studies, evaluating the association between MPV and mortality after acute myocardial infarction (AMI), reported that mortality after AMI was higher in patients with higher MPV values. Moreover, the authors found a greater risk of restenosis after angioplasty in infarcted patients with high MPV. Accordingly, MPV is also a useful parameter in predicting the outcome of these patients [19].

The increase in MPV is not only related to CAD but also numerous other vascular diseases. Indeed, a recent meta-analysis of 27 studies that explored MPV in stroke showed significantly higher MPV values in patients with stroke than in healthy controls. Besides, this increase persists even several months after the acute event [20]. In another study, including 3134 patients with a history of cerebrovascular disease, the authors showed an 11% increase in the relative risk of developing an acute stroke for each 1-fl of MPV increase [21].

Likewise, the large cross-sectional study National Health and Nutrition Examination Survey (NHANES), conducted on 6354 participants from 1999 to 2004, showed a higher prevalence of peripheral artery disease (PAD), defined for ankle–brachial index values ≤0.9, in patients with elevated MPV. The authors also showed that the correlation between MPV and PAD remained significant even after correction of possible confounding factors, suggesting a role of MPV as an independent risk factor for the development of PAD [22].

### 3.2. Venous Diseases

MPV has also been related to venous diseases. Pyo and colleagues showed in their meta-analysis that a high MPV correlates with the presence and grade of varicocele, suggesting a role for this marker in the diagnosis and staging of this pathology [23]. The increase in MPV in these patients could be a consequence of the vascular damage induced by varicose disease, which favors the activation of platelets. This theory seems to be confirmed by the fact that the surgical treatment of the disease is associated with a normalization of MPV values [24].

## 4. Mean Platelet Volume and Erectile Dysfunction

Erection is a complex mechanism in which the vascular component plays a major role. In fact, during erection, the arteries dilate, the sinusoidal spaces expand, and consequently, the veno-occlusive mechanism is activated by the compression of the veins between the blood-filled sinusoids and the albuginea tunic [25]. Given the relevance of high MPV values in vascular diseases and that erection is mainly a vascular event, several studies have been conducted to evaluate the correlation between MPV and ED. In a retrospective study, Aldemir and colleagues showed significantly higher MPV values in patients with ED than in the control group (10.7 ± 1.0 vs. 9.72 ± 1.5) [26]. Similarly, another study involving 230 patients, showed that 130 patients with ED had significantly higher MPV and platelet count than the 100 control patients. Moreover, the logistic regression analysis, performed after adjusting for factors known to be associated with ED (such as diabetes, hypertension, and dyslipidemia), highlighted that MPV is an independent risk factor for the development of ED [27]. Conversely, one study found no significant differences in MPV values between patients with ED and healthy controls [28].

When only patients with vasculogenic ED diagnosed by penile echo-color Doppler ultrasound (PCDU) are considered, the negative role of high MPV levels on erectile function becomes even clearer. Indeed, Ciftci and colleagues not only found higher MPV values in patients with vasculogenic ED compared to healthy controls [29] but in a second study they also showed that MPV values were higher in patients with vasculogenic ED compared to patients with other forms of ED (particularly patients with post-prostatectomy ED) [30]. The role of MPV in the vascular etiopathogenesis of ED was confirmed by two meta-analyses. A meta-analysis of seven studies including 1319 patients confirmed that patients with ED have higher MPV values than controls and, particularly, patients with vasculogenic ED had significantly higher values compared to both healthy controls and patients with non-vascular ED [31]. A subsequent meta-analysis of 13 studies including 1595 patients with ED and 967 healthy men reached the same conclusion [32].

Only two studies separately assessed MPV levels in arterial and venous ED patients with conflicting results. Indeed, Wang and colleagues showed that patients with arteriogenic ED have significantly higher MPV values than patients with venous ED and healthy controls, with a negative correlation between MPV and peak systolic velocity (PSV) 10 min after intracavernosal drug administration. They also established an MPV cut-off of 9.65 fl for arterial ED diagnosis, with a specificity of 91.7% [33]. Conversely, a study conducted on 30 diabetic patients with ED did not find any difference between the different vasculogenic ED subtypes, thus suggesting that the increase in MPV in diabetic patients is correlated with vasculogenic ED regardless of the type of dysfunction (arterial, venous, or mixed) [34].

Consequently, further studies are needed to better clarify whether MPV is associated with vasculogenic ED in general or more specifically with its arteriogenic form.

Different studies have focused exclusively on assessing the role of MPV on arteriogenic ED. Again, studies agreed on the negative role of MPV on erectile function. Indeed, Bayraktar and colleagues showed significantly higher MPV values in 70 patients with arteriogenic ED compared to 50 sexually active controls with normal IIEF scores (11.27 ± 0.56 vs. 9.8 ± 0.91) [35]. Similarly, a study including 36 patients with arteriogenic ED, diagnosed for PSV values < 30 cm/s at the PCDU, showed that MPV values were significantly higher compared to 32 controls with non-vascular ED. The authors concluded that the MPV value has a positive predictive value of 82% for the development of arteriogenic ED [36].

If the literature agrees that high MPV values correlate with ED and in particular with its arterial etiopathogenesis, the evidence on the role of MPV in establishing the severity of ED is conflicting. In a previous study, we assessed the MPV values in patients with different arteriogenic ED degrees of severity, diagnosed by dynamic PCDU, by comparing them with each other and with a group of control ED patients without arterial dysfunction. All patients enrolled in this study did not have the classical cardiovascular risk factors. The results showed that MPV levels were significantly higher in patients with arteriogenic ED than in controls and that MPV values increased significantly as the severity of ED increased. A similar trend was found for the expression levels of αVβ3, involved, as said before, in the first phase of platelet activation. Patients with arteriogenic ED had a higher αVβ3 expression than controls and patients with a more severe form of arteriogenic ED had significantly higher expression than those with milder forms. The correlation analysis showed a positive link between MPV and αVβ3, and a negative correlation between MPV and PSV [37]. Similarly, a study on 434 patients including 312 with ED diagnosed using the IIEF-5 questionnaire and 122 without ED (score IIEF-5 > 21) showed not only that MPV values are higher in ED patients than the controls, but also that MPV increases as the severity of ED increases [38]. Likewise, El Taieb and colleagues found that MPV increases with the falling of the IIEF score [34]. Conversely, other studies have not shown a correlation with the severity of ED [28,29]. Guo and colleagues reported that, although MPV was significantly higher in patients with moderate and severe forms of ED than in healthy patients, no negative correlations were found between IIEF scores and MPV values considering individual ED severity groups. In contrast, the negative correlation between MPV and IIEF scores was found when the entire population was considered. This suggests a role for MPV in predicting the development of ED, but not on the severity of the condition [39]. Probably the lack of agreement between the studies on this aspect may be ascribed to the heterogeneity of the patients enrolled in the different studies. Further studies on the specific arteriogenic subtype of ED could better clarify this aspect. Table 1 summarizes the characteristics of the patients enrolled and the results of the studies that evaluated the role of MPV in patients with ED.

## 5. Limits

Although based on the present literature the correlation between MPV and arteriogenic ED is quite evident, the limitations present in these studies must be considered. Indeed, since all of them are observational studies (case-control), it is not possible to establish a cause-effect relationship between high MPV and ED, just as it is not possible to establish a temporal relationship between this parameter and ED. Further, many studies have been conducted on a low number of patients [26,29,30,33,34,37,39]. Even for the diagnosis of ED, the studies are heterogeneous. In fact, in several of them, the diagnosis was made only by the administration of the IIEF-5 questionnaire, thus it is impossible to exclude the presence of non-vasculogenic ED [26,27,28,38,39]. Furthermore, the ED severity was evaluated by PCDU only in our study [37], whereas the others based the severity assessment on the IIEF-5 score [27,28,29,30,34,38,39].

Other limits to consider are of analytical and pre-analytical order. Not all the studies have reported the time between blood withdrawal and the moment when the sample was analyzed. This is an important issue since the sample should be analyzed within one hour to prevent platelet activation. Furthermore, it should not be frozen because low temperatures increase platelet volume [40]. In almost all studies, blood was collected in tubes containing ethylen-diamine-tetra-acetic acid (EDTA), which is associated with an increase in platelet volume in a time-dependent manner [40]. Even the method of measuring platelets, being different according to the laboratories, does not allow an adequate standardization of the reference parameters [40]. Moreover, in all studies, the difference in MPV values between ED patients and controls is very small and could also be due to the inter-individual variability of the sample [41].

Finally, only a few studies ruled out the cardiovascular risk factors that are associated with increased MPV values and ED prevalence [28,29,34,35,36]. MPV increases in smokers compared to non-smokers, though this is particularly true in postmenopausal women [42] and decreases with smoking cessation [43]. Moreover, hypertension also appears to correlate with an increased MPV [42]. Gang and colleagues reported a higher prevalence of hypertension in patients with higher MPV values that remains significant even when confounding factors, such as age, sex, waist circumference, drinking status, platelet count, and creatinine levels, were excluded from the analysis. This would suggest a role of MPV also in the development of hypertension [44].

As for diabetes mellitus, hyperglycemia and insulin resistance associated with this disease (at least for type 2) induce systemic inflammation, oxidative stress, and reduction in the bioavailability of nitric oxide (NO) consequent to endothelial damage. All these conditions favor the activation of platelets resulting in the release of prothrombotic and proinflammatory cytokines. Furthermore, hyperglycemia and oxidative stress favor the glycation of some membrane proteins essential for a proper platelet function, such as P-selectin and Glycoprotein IIb/IIIa, making them more susceptible to bind their ligands [45]. In a recent study, Inoue and colleagues showed that MPV values increased significantly in diabetic and pre-diabetic patients compared to non-diabetics, with a positive correlation between MPV and fasting glucose and glycated hemoglobin. This positive correlation is also maintained by adjusting for confounding factors and is further proven by MPV reduction when glycometabolic parameters ameliorate. Moreover, they showed a positive correlation between MPV and the cardio–ankle vascular index, a marker of vascular stiffness, and therefore of atherosclerosis, thus suggesting a role of MPV in predicting the macrovascular complications of diabetes and the progression of atherosclerotic disease [46]. Finally, obesity and dyslipidemia have also been reported as associated with higher MPV values [42].

Other conditions associated with ED, CVD, and increased MPV are vitamin D deficiency and thyroid dysfunction. Culha and colleagues showed that patients with severe forms of ED have significantly lower vitamin D levels than patients with mild forms. They also highlighted a negative correlation between vitamin D and MPV values, suggesting that vitamin D deficiency is associated with high MPV levels [47]. The literature has shown a role of vitamin D deficiency in the etiopathogenesis of ED and CVD through various mechanisms, such as endothelial dysfunction, inflammation, oxidative stress, and alteration of glucose homeostasis [48]. Two mechanisms would explain the increase in MPV in the patient with vitamin D deficiency: endothelial dysfunction and the inflammatory state induced by vitamin D deficiency expressed as an increase in pro-inflammatory cytokines such as IL-6 and TNFα [49]. As for thyroid dysfunction, high TSH levels are known to be associated with a decrease in endothelial availability of NO, a key molecule for erectile function [50]. Similarly, the increase in TSH levels in patients with subclinical hypothyroidism is associated with poorer cardiac performance and a higher CAD rate, due to the increased systemic vascular resistance and the reduced endothelial-mediated vasorelaxation that are associated with this condition [51]. Furthermore, shreds of evidence in the literature show an increase in MPV in patients with subclinical hypothyroidism [52]. This suggests that subclinical hypothyroidism can contribute to ED and CVD directly through the reduction of NO and indirectly through the increase in MPV. Accordingly, in a recent study, we have shown how treatment with levothyroxine reduces MPV levels and improves erectile function in patients with subclinical hypothyroidism compared to patients who are not undergoing treatment [53].

If by one hand the increase in MPV values in diseases increasing the cardiovascular risk indicates a common underlying pathophysiological mechanism [32], on the other, it is not possible to say whether MPV is a direct risk factor for CAD and ED or only an indirect marker [17]. Indeed, some authors dispute the usefulness of MPV for the early diagnosis of vascular diseases, starting from the assumption that the high MPV is a consequence of the vessel occlusion rather than the cause. The release of thrombin and other molecules during the thrombotic event would increase the size of the platelets. Furthermore, the increase in MPV would be due to the release of young platelets (larger in diameter than the mature ones) stimulated by the increase in thrombopoietin levels following the consumption of platelets during the thrombotic event [41]. However, the few studies conducted on patients without cardiovascular risk factors and the regression analyses excluding these factors performed in other studies seem to indicate that MPV may have an independent role in determining both ED [27,29,30,37,38] and CVD [54]. Certainly, further prospective studies performed on larger population samples, evaluating whether ED patients with higher MPV have a greater risk of developing CVD than the counterpart with normal MPV levels, are needed to better understand this aspect. Moreover, studies evaluating the association between MPV and other risk factors known to be associated with endothelial dysfunction and cardiovascular risk would also be useful. For example, flow-mediated dilation (FMD) is a parameter that correlates both with the risk of CVD development and the presence of ED [55], however studies that have evaluated an association between this parameter and MPV are lacking. Other markers of endothelial damage are the endothelial progenitor cells (EPCs) and the endothelial microparticles (EMPs). EPCs are able to differentiate into mature endothelial cells, being an expression of the ability to repair endothelial damage. However, there are several EPC and EMP phenotypes and we have shown that the EPC phenotype CD45_neg_/CD34_pos_/CD144_pos_ and the EMP phenotype CD45_neg_/CD144_pos_/Annexin V_pos_ are dysfunctional and that their levels are significantly higher in patients with arteriogenic ED [56]. It might therefore be useful to evaluate whether MPV also correlates with increased levels of dysfunctional phenotypes of EPCs and EMPs. Table 2 lists the characteristics and limits of the studies included in this review.

## 6. Conclusions

MPV, albeit with its limitations, is a simple and cheap parameter to evaluate. Therefore, it can be used to identify the patients with ED who should undergo PCDU, which represents the gold standard for the diagnosis of arteriogenic ED [37]. Since, as above-mentioned, arteriogenic ED precedes CAD by several years [3], MPV promises to be an early marker of vascular dysfunction capable of identifying those patients who would benefit from a long-term monitoring with consequent prevention of major adverse cardiovascular events. Furthermore, given the association between MPV and other parameters not routinely evaluated in patients with ED, but which have a role in causing endothelial dysfunction (such as hypovitaminosis D and subclinical hypothyroidism), the finding of high MPV could favor a thorough laboratory evaluation of these patients and consequently the correction of alterations that normally could go unnoticed.

## Figures and Tables

**Table 1:** Sample size, population characteristics, and results of studies evaluating the role of mean platelet volume (MPV) in patients with erectile dysfunction (ED) *.

Authors	N of Cases	N of Controls	MPV of Cases	MPV of Controls	Mean Age of Cases	Mean Age of Controls	Study Result
Aldemir, 2016	57	59	10.7 ± 1	9.72 ± 1.5	49.7 ± 12	49.7 ± 10.6	Mean MPV values were significantly higher in patients with ED than controls
Bayratar, 2017	70	50	11.27 ± 0.56	9.8 ± 0.91	48.1 ± 11.7	47.6 ± 12.3	Mean MPV values were significantly higher in patients with vasculogenic ED than controls
Cannarella, 2020	20 (treated) 20 (not treated)	/	12.3 ± 0.3 11.58 ± 0.7	/	61.2 ± 8.8 60.3 ± 5.6	/	Treatment with LT4 improves erectile function and decreases MPV levels
Cifti, 2013	50	40	7.49 ± 1.4	6.85 ± 1.2	53.7 ± 12.39	53.85 ± 9.5	MPV values were significantly higher in ED patients than controls group. No difference in MPV levels between patients with severe ED and those with mild ED
Cifti, 2014	50 (vasculogenic ED) 30 (post-prostatectomy ED)	40	7.49 ± 1.4 6.43 ± 1.19	6.85 ± 1.2	53.7 ± 12.39 54.6 ± 11.4	53.85 ± 9.5	MPV values in a vasculogenic ED group of patients were significantly higher than in the patients with post-prostatectomy ED group and controls group. No difference in MPV values between post-prostatectomy group and control groups
Culha, 2018	41 (mild ED) 49 (severe ED)	/	10.07 ± 0.85 10.79 ± 1.03	/	39.8 ± 8.51 42.63 ± 8.37	/	Vitamin D levels were significantly lower in the patients with severe ED group than the mild ED group with a negative correlation between MPV and vitamin D levels. Negative correlation between MPV and IIEF score. Negative correlation between vitamin D levels and IIEF-5
El Taieb, 2018	30	20	9.81 ± 0.7	7.98 ± 0.9	54.43 ± 9.8	41.83 ± 12.9	MPV values were significantly higher in ED patients than the control group. Increase in MPV values increased as the severity of ED worsened according to the IIEF-5 score. No difference in MPV values between the different subtypes of ED was reported
Guo, 2016	118 (mild ED) 120 (severe ED)	120	9.24 ± 0.7 9.71 ± 0.8	8.56 ± 0.62	37.58 ± 6.64 38.96 ± 6.24	36.65 ± 6.11	Progressive increase in MPV as ED worsens. However, correlation analysis between MPV and IIEF-5 did not show any significant correlation
La Vignera, 2014	5 (severe arteriogenic ED) 6 (moderate arteriogenic ED) 4 (mild arteriogenic ED)	10	/	/	65 ± 2 66 ± 3 64 ± 2	65 ± 4	Significantly higher MPV values in arteriogenic ED patients than controls with progressive increase in MPV values in patients as PSV values decrease (negative correlation between MPV and PSV). Significantly higher αVβ3 in arteriogenic ED patients than controls with a direct correlation between MPV and αVβ3
Otunctemur, 2015	180	120	8.51 ± 1	8.16 ± 0.94	55.62 ± 8.9	54.19 ± 4.1	Mean MPV values were significantly higher in patients with ED than controls
Ren, 2016	/	/	9.1	9.512	/	/	MPV values were significantly higher in patients with ED than controls and in particular in those with vasculogenic ED
Senturk, 2018	102	203	9.49 ± 1.66	9.39 ± 1.56	/	/	MPV did not differ significantly. No difference in MPV values between cases and controls
Sonmez, 2016	36 (arteriogenic ED) 32 (nonvasculogenic ED)	/	9.93 ± 1.01 8.82 ± 0.92	/	53.8 ± 11.4 51.2 ± 10.8	/	MPV was significantly higher in arteriogenic ED than non-vascologenic ED
Tangal, 2018	62 (severe ED) 78 (moderate ED) 80 (mild to moderate ED) 92 (mild ED)	122	9.5 ± 0.7 9.3 ± 0.6 8.7 ± 0.6 8.4 ± 0.4	8.2 ± 0.5	/	/	MPV values were significantly higher in patients with ED than controls. A positive correlation between MPV values and ED severity was found
Wang, 2019	99 (arteriogenic ED) 37 (venous ED)	60	9.59 ± 0.98 8.9 ± 0.55	8.91 ± 0.57	29.41 ± 8.06 28.78 ± 7.17	29.51 ± 6.13	MPV was significantly higher in the arteriogenic ED group than the venous ED group and control group. No difference in MPV values between venous ED group and control group. Negative correlation between MPV and 10-min PSV
Yang 2019	1595	967	/	/	/	/	MPV values were significantly higher in patients with ED than controls, in particular in those with vasculogenic ED

* No information on the ethnicity of the population.

**Table 2:** Main characteristics and limits of the studies evaluating the role of MPV in patients with erectile dysfunction.

Authors	Study Design	Diagnosis of Erectile Dysfunction	Assessment of the Severity of Erectile Dysfunction	Time between Blood Collection and Analysis of the Sample	Limits
Aldemir, 2016	Case-control	IIEF-5	/	Immediately	Small sample; blood collected in tubes containing EDTA; erectile dysfunction evaluated only with IIEF-5; failure to exclude other risk factors associated with high MPV such as smoking, hypertension, and diabetes
Bayratar, 2017	Case-control	PCDU	IIEF-5	<1 h	Small sample; blood collected in tubes containing EDTA
Cannarella, 2020	Case-control	PCDU	IIEF-5	Not specified	Small sample; the anticoagulant inside the tubes and the time elapsed from blood collection to analysis are not specified; study performed on patients with SCH
Cifti, 2013	Case-control	PCDU	IIEF-5	Immediately	Small sample; blood collected in tubes containing EDTA
Cifti, 2014	Case-control	PCDU	IIEF-5	Immediately	Small sample; blood collected in tubes containing EDTA
Culha, 2018	Case-control	IIEF-5	IIEF-5	Not specified	Small sample; the anticoagulant inside the tubes and the time elapsed from blood collection to analysis are not specified; erectile dysfunction evaluated only with IIEF-5
El Taieb, 2018	Case-control	PCDU	IIEF-5	Not specified	Small sample; the anticoagulant inside the tubes and the time elapsed from blood collection to analysis are not specified; study performed on diabetic patients; patients significantly older than controls
Guo, 2016	Case-control	IIEF-5	IIEF-5	<1 h	Blood collected in tubes containing EDTA; erectile dysfunction evaluated only with IIEF-5; failure to exclude other risk factors associated with high MPV such as smoking and hypertension; patients with both mild and severe ED significantly older than controls
La Vignera, 2014	Case-control	PCDU	PCDU, IIEF-5	Immediately	Small sample; blood collected in tubes containing EDTA
Otunctemur, 2015	Case-control	IIEF-5	IIEF-5	Not specified	The anticoagulant inside the tubes and the time elapsed from blood collection to analysis are not specified; erectile dysfunction evaluated only with IIEF-5; failure to exclude other risk factors associated with high MPV such as smoking and hypertension
Ren, 2016	Meta-analysis	/	/	/	Meta-analysis performed only on a few studies with a relatively small sample; no analysis of the association between MPV and severity of erectile dysfunction; no evaluation of the association between MPV and cardiovascular risk in patients with ED
Senturk, 2018	Case-control	IIEF-5	/	Not specified	The anticoagulant inside the tubes and the time elapsed from blood collection to analysis are not specified; erectile dysfunction evaluated only with IIEF-5; failure to exclude other risk factors associated with high MPV such as smoking and dyslipidemia
Sonmez, 2016	Case-control	PCDU	IIEF-5	Immediately	Small sample; blood collected in tubes containing EDTA
Tangal, 2018	Case-control	IIEF-5	IIEF-5	<1 h	Blood collected in tubes containing EDTA; erectile dysfunction evaluated only with IIEF-5; failure to exclude other risk factors associated with high MPV such as smoking, hypertension, diabetes, and dyslipidemia
Wang, 2019	Case-control	PCDU	/	<1 h	Small sample; blood collected in tubes containing EDTA; failure to exclude other risk factors associated with high MPV such as smoking and hypertension
Yang 2019	Meta-analysis	/	/	/	Heterogeneity of included studies; no analysis of the association between MPV and severity of erectile dysfunction; no evaluation of the association between MPV and cardiovascular risk in patients with ED

## Abbreviations

αVβ3 = vitronectin receptor; CAD = coronary artery disease; CVD = cardiovascular disease; ED = erectile dysfunction; EDTA = ethylen-diamine-tetra-acetic acid; IIEF = International Index of Erectile Function; MPV = mean platelet volume; NO = nitric oxide; PAD = peripheral artery disease; PCDU = penile color Doppler ultrasound; PF4 = platelet factor 4; RANTES = regulated upon activation, normal T cell expressed and secreted; VD = vitamin D; VDD = vitamin D deficiency; VWF = von Willebrand factor.
